# TECRR: a benchmark dataset of radiological reports for BI-RADS classification with machine learning, deep learning, and large language model baselines

**DOI:** 10.1186/s12911-024-02717-7

**Published:** 2024-10-24

**Authors:** Sadam Hussain, Usman Naseem, Mansoor Ali, Daly Betzabeth Avendaño Avalos, Servando Cardona-Huerta, Beatriz Alejandra Bosques Palomo, Jose Gerardo Tamez-Peña

**Affiliations:** 1 School of Engineering and Sciences, Tecnológico de Monterrey, Monterrey, 64849 Nuevo Leon Mexico; 2 School of Computing, Macquarie University, Sydney, 2109 NSW Australia; 3 School of Medicine, Tecnológico de Monterrey, Monterrey, 64849 Nuevo Leon Mexico

**Keywords:** BI-RADS classification, Breast radiological reports, TF-IDF, Word2vec, NLP, ML

## Abstract

**Background:**

Recently, machine learning (ML), deep learning (DL), and natural language processing (NLP) have provided promising results in the free-form radiological reports’ classification in the respective medical domain. In order to classify radiological reports properly, a high-quality annotated and curated dataset is required. Currently, no publicly available breast imaging-based radiological dataset exists for the classification of Breast Imaging Reporting and Data System (BI-RADS) categories and breast density scores, as characterized by the American College of Radiology (ACR). To tackle this problem, we construct and annotate a breast imaging-based radiological reports dataset and its benchmark results.

The dataset was originally in Spanish. Board-certified radiologists collected and annotated it according to the BI-RADS lexicon and categories at the Breast Radiology department, TecSalud Hospitals Monterrey, Mexico. Initially, it was translated into English language using Google Translate. Afterwards, it was preprocessed by removing duplicates and missing values. After preprocessing, the final dataset consists of 5046 unique reports from 5046 patients with an average age of 53 years and 100% women. Furthermore, we used word-level NLP-based embedding techniques, term frequency-inverse document frequency (TF-IDF) and word2vec to extract semantic and syntactic information. We also compared the performance of ML, DL and large language models (LLMs) classifiers for BI-RADS category classification.

**Results:**

The final breast imaging-based radiological reports dataset contains 5046 unique reports. We compared K-Nearest Neighbour (KNN), Support Vector Machine (SVM), Naive Bayes (NB), Random Forest (RF), Adaptive Boosting (AdaBoost), Gradient-Boosting (GB), Extreme Gradient Boosting (XGB), Long Short-Term Memory (LSTM), Bidirectional Encoder Representations from Transformers (BERT) and Biomedical Generative Pre-trained Transformer (BioGPT) classifiers. It is observed that the BioGPT classifier with preprocessed data performed 6% better with a mean sensitivity of 0.60 (95% confidence interval (CI), 0.391-0.812) compared to the second best performing classifier BERT, which achieved mean sensitivity of 0.54 (95% CI, 0.477-0.607).

**Conclusion:**

In this work, we propose a curated and annotated benchmark dataset that can be used for BI-RADS and breast density category classification. We also provide baseline results of most ML, DL and LLMs models for BI-RADS classification that can be used as a starting point for future investigation. The main objective of this investigation is to provide a repository for the investigators who wish to enter the field to push the boundaries further.

**Supplementary Information:**

The online version contains supplementary material available at 10.1186/s12911-024-02717-7.

## Introduction

Breast cancer is the leading type of cancer diagnosed in women worldwide [1]. It poses a significant public health concern and carries a substantial economic burden. Early breast cancer diagnosis is widely recognized as a critical factor in reducing mortality rates [2]. Mammography screening is a recommended method for the early detection of breast cancer in average-risk women [3, 4]. To standardize terminology and categorize results for each breast imaging modality (i.e., mammography, ultrasound, MRI, and DBT), the ACR developed the BI-RADS. This system consists of seven categories, ranging from (0 through 6), where 0 is inconclusive, 1 is negative, 2 is benign, 3 is probably benign, 4 is suspicious, 5 is highly suggestive of malignancy, and 6 is known biopsy-proven malignancy. In addition to clinical settings, the BI-RADS system is a quality assurance tool in research [5].

The manual method is the state-of-the-art (SOTA) of extracting information from free text, which is costly, error-prone, and time-consuming, especially when dealing with large datasets. Free-form text is a way of writing radiology reports without a template, which can be more expressive and efficient but also more challenging to standardize and interpret [6–8]. To overcome these challenges, different NLP methods have been developed. These methods have revealed promising results in extracting crucial information from radiological reports, enabling easy access to appropriate information to be analyzed for various clinical applications [9–11].

Recently, there has been a great interest in using AI algorithms to increase the accuracy of BI-RADS prediction from breast imaging-based radiological reports [12–17]. Nonetheless, the development and evaluation of such AI algorithms require quality and large-scale datasets, including radiological reports, that are very rare. Therefore, we are annotating and releasing a novel dataset. This work presents a new dataset on breast imaging-based radiological reports and its baselines. The dataset contains over 5,000 radiological reports from 2D mammography, 3D mammography, and breast ultrasound (US). The data was collected from TecSalud Hospitals (Monterrey, Mexico). The dataset was translated into English from Spanish and preprocessing techniques were applied to remove the duplicates and missing values with the consultation of radiologists. We tried different word embedding techniques to vectorize the radiological reports in order to extract the syntactic and semantic meaning that is subsequently helpful for BIRADS classification. We also used different ML, DL and LLMs architectures to provide baselines for classifying BI-RADS. This dataset and baselines can serve as a valuable resource for researchers working on AI algorithms for breast imaging-based radiological reports and can contribute to enhancing the accuracy and efficacy of breast cancer diagnosis.

## Methods

Breast imaging is the angular stone for early detection and diagnosis of breast cancer and other breast-related conditions [4, 18]. Currently, the reporting system for breast imaging studies differs significantly among radiologists and institutions and is predominantly based on traditional free-text reporting [19]. However, structured reporting is being promoted to improve reporting in radiology, which would benefit radiological, clinical practice, and data mining in an ongoing project. Meanwhile, we have substantial pre-existing data that requires retrospective analysis. To address this issue, the ACR developed the BI-RADS, a standardized system for describing and communicating breast imaging results [20]. The BI-RADS provides a common language and a classification scheme for mammography, ultrasound, MRI, and DBT of the breast, as well as guidance for follow-up and outcome monitoring. The BI-RADS also enables radiologists to perform quality assurance and improvement through medical audits and data analysis.

This paper presents a new breast imaging-based radiological reports dataset that follows the BI-RADS framework and covers multiple imaging modalities. Our dataset contains over 5,000 radiology reports from different patients, annotated by board-certified radiologists according to the BI-RADS lexicon and categories [21, 22]. The BI-RADS category 0 was assigned when additional evaluation was required. The BI-RADS category 1, commonly known as negative, was assigned when there was no evidence of malignancy in either breast, and they are symmetrical with no masses, architectural distortion, or suspicious calcifications. The BI-RADS category 2, i,e benign, was assigned when a benign finding did not require further evaluation or follow-up, such as a simple cyst, fibroadenoma, intramammary lymph node, or benign calcifications. The BI-RADS category 3 (probably benign) was assigned when there was less than a 2% chance of being malignant and can be safely monitored with short-interval follow-up imaging, such as a probably benign mass, focal asymmetry, or clustered microcysts. The BI-RADS category 4 (suspicious) was assigned when there was a 2-94% chance of being malignant, and biopsy should be considered, such as a spiculated mass, architectural distortion, or suspicious calcifications. The BI-RADS category 5 (highly suggestive of malignancy) was assigned when there was a chance of greater than 95% of being malignant and appropriate action should be taken. The BI-RADS category 6 (known biopsy-proven malignancy) was assigned when there was histological confirmation of malignancy in the breast before any treatment had been initiated [23, 24]. We use our dataset to benchmark several ML, DL and LLMs methods on the BI-RADS correct category classification. We also provide clinical applications and insights based on our dataset and results. This dataset can be requested to use.

We introduce a novel and comprehensive breast imaging-based radiological reports dataset that adheres to the BI-RADS standard and covers multiple modalities reports. Using our dataset, we propose SOTA NLP pipeline-based ML, DL and LLMs benchmarks on the BI-RADS correct category classification, demonstrating its potential for advancing research in both domains.

### Data

The dataset was collected from the Breast Radiology department, TecSalud hospitals (Monterrey, Mexico). It was approved by the ethical committee to give access to the data upon reasonable request. This dataset comprises digital mammography (DM), digital breast tomosynthesis (DBT), and breast ultrasound (US) based radiological reports. All the reports were anonymized. These reports were originally in Spanish and collected from January to December 2014. The reports were then translated online using Google Translate and were verified by the radiologist, who is better aware of English and Spanish. The reports were created and evaluated by (*n*=2) trained radiologists. There were (*n*=7904) actual entries in our dataset. However, after preprocessing, i.e., removing reports with missing values, duplicate records, and BI-RADS categories (0-inconclusive) and (6-biopsy proven), we were able to utilize 5,046 unique reports with an average age of 53 years and 5046 (100.0%) women from 7,904 reports for the final model building and evaluation. The overview of the dataset is given in Table 1.

### Preprocessing

**Fig. 1:**
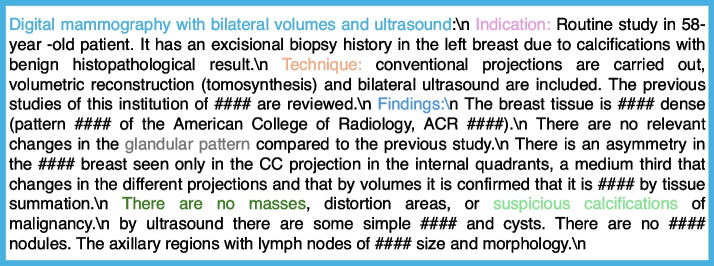
Breast imaging based radiological report (This is a fabricated report for demonstration purpose)

**Fig. 2:**
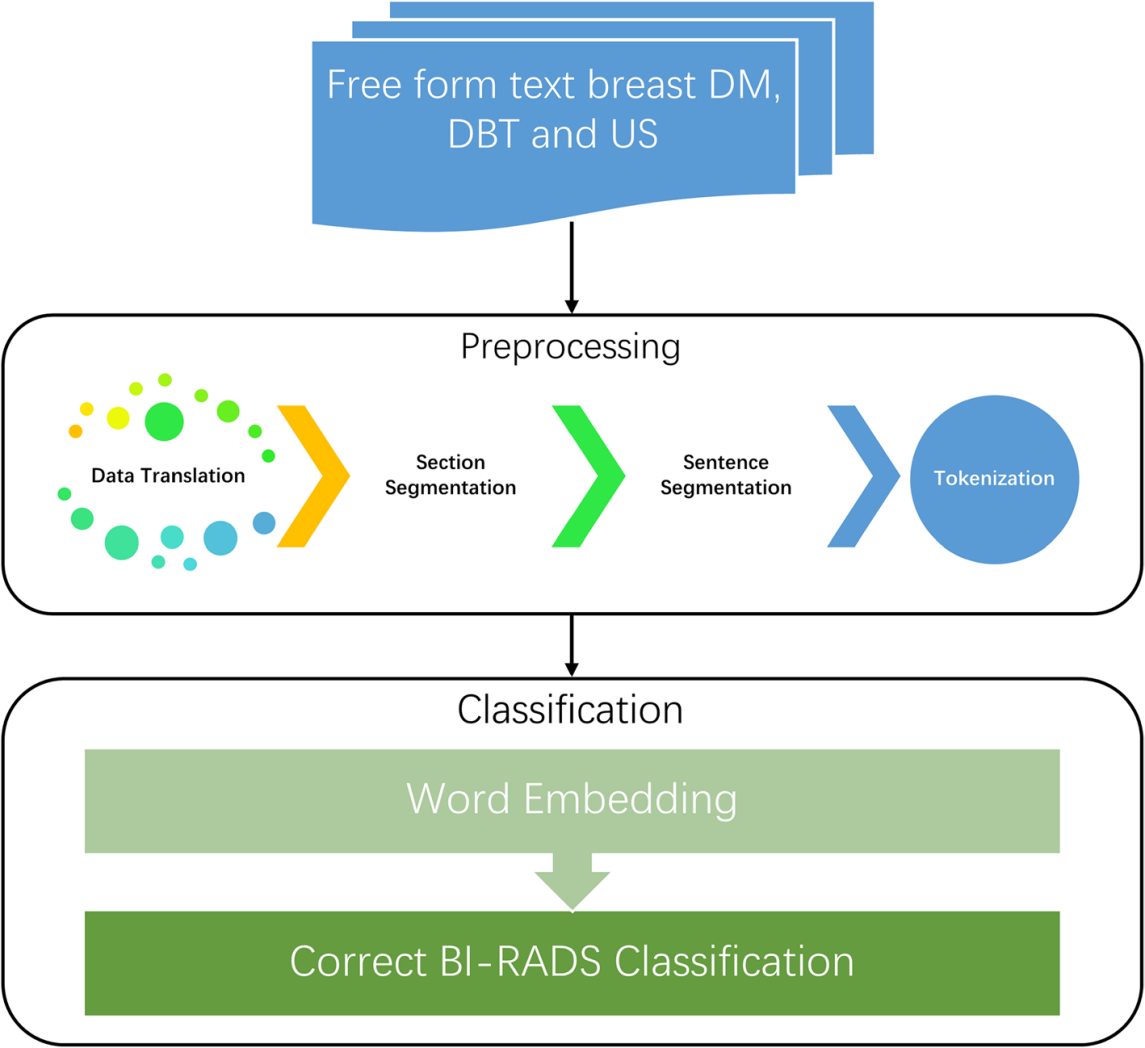
NLP workflow for preprocessing reports and BI-RADS classification

Our original dataset was in the Spanish language. The dataset was translated online using Google Translate. The single entry of the patient consists of a brief clinical indication of the study, and personal risk history, both imaging description, findings, and diagnostic impression. As shown in Fig. 1, each original DM, DBT, and breast US report consists of multiple paragraphs. We developed an NLP-based pipeline to extract associated imaging features for the final BI-RADS category classification. The pipeline consists of various analysis filters for different clinical and linguistics tasks, like sentence segmentation, sentence detection, tokenization, detection of concepts, and data normalization. The pipeline is described in Fig. 2.

### Data curation

Data curation was conducted on the TECRR dataset to ensure its quality and suitability for analysis. The process involved several key steps: first, all references to doctors were removed to maintain privacy and confidentiality. Duplicated structured radiological reports were then identified and eliminated to prevent redundant information. Dates within the reports were removed to minimize potential bias related to temporal factors, and any non-radiological information was excluded to focus solely on the relevant radiological data. Additionally, density scores and BI-RADS scores were standardized across the dataset to enhance comparability and consistency. Finally, the structured reports were segmented into sections, including the main body, patient description, and report conclusion.

### Word embedding techniques

We employed word-level NLP-based embedding techniques such as TF-IDF [25] and word2vec [26] to extract semantic and syntactic information from the free-form text of radiological reports. TF-IDF is a statistical measure that evaluates the importance of words based on their frequency in a document and their rarity across the entire corpus. It assigns higher weights to words that frequently occur in a document but are rare in the corpus. The formula for TF-IDF is:

1 $$\begin{aligned} \text {tf-idf}(w,d,D) = \text {tf}(w,d) \times \text {idf}(w,D) \end{aligned}$$

where $w$ is a word, $d$ is a document, and $D$ is the collection of documents. The term frequency, $tf(w,d)$, represents how often word $w$ appears in document $d$, while the inverse document frequency, $idf(w,D)$, is calculated as:

2 $$\begin{aligned} \text {idf}(w,D) = \textrm{log} \frac{|D|}{|d \in D : w \in d|} \end{aligned}$$

Here, $|D|$ is the total number of documents, and $|d \in D : w \in d|$ represents the number of documents that contain the word$w$. TF-IDF increases for words that appear frequently in a specific document but are rare across the collection, thus emphasizing distinctive terms. Word2vec [26], on the other hand, is a neural network-based model that generates word embeddings-vector representations of words that capture both semantic and syntactic relationships. Word2vec uses either a skip-gram model, which predicts context words given a target word, or a continuous bag-of-words (CBOW) model, which predicts the target word from its surrounding context. The word2vec skip-gram objective is to maximize the likelihood of context words given a target word:

3 $$\begin{aligned} \text {word2vec}(w,c,D) = \textrm{log} \frac{\exp \left(v_w^T v_c\right)}{\sum _{w' \in D} \exp \left(v_{w'}^T v_c\right)} \end{aligned}$$

where $w$ is the target word, $c$ is a context word, $D$ is the vocabulary, and $v_w^T v_c$ represents the dot product of the word embeddings of $w$ and $c$. This formula can be interpreted as the probability of observing a context word given a target word, normalized by a softmax function over the vocabulary. The word embeddings are learned by maximizing this probability over all word-context pairs in the corpus.

### State-of-the-art models

We compared the performance of ML, DL and LLMs models. The ML models are KNN, SVM, NB, RF, AdaBoost, GB and XGBoost; the DL model is LSTM [27]; and the LLMs models are BERT [28] and BioGPT [29]. LSTM networks capture long-term dependencies in sequential data, making them suitable for modelling temporal patterns in radiological reports. BERT, particularly the “bert-base-uncased” model, leverages a bidirectional transformer architecture to capture context from both directions in a sentence. This version of BERT consists of 12 layers, 12 attention heads, and approximately 110 million parameters, focusing on uncased English text. Furthermore, BioGPT, a specialized transformer model for biomedical text generation, is employed for tasks such as medical report generation and knowledge extraction. The tokenization for BioGPT is handled using the BioGPT Tokenizer, which processes biomedical vocabulary and ensures precise tokenization of medical terminology. These advanced models capture more complex contextual and semantic relationships than traditional methods, enhancing tasks such as information retrieval, classification, and summarization of radiological reports. These methods enable the extraction of key information from the often unstructured text of radiological reports, which may contain specialized medical terminology [30]. The semantic relationships between words, captured by these embeddings, facilitate tasks such as information retrieval, classification, and summarization of radiological reports [31].

### Evaluation metrics

We report sensitivity and accuracy with 95% CI. A 95% CI indicates that the model is confident that, in 95 out of 100 cases, the true prediction will fall within the upper and lower bounds of the interval. Sensitivity or recall is the proportion of true positives among all positive cases. Sensitivity is a measure of how well a test can correctly identify true positives, i.e., cases of breast cancer. Sensitivity is good for BI-RADS prediction because it can help reduce the number of false negatives, i.e., cases of breast cancer that are missed by the test. A high sensitivity means that the test can capture most of the breast cancer cases and avoid unnecessary delays in diagnosis and treatment [32]. The formula for the sensitivity is given below,

4 $$\begin{aligned} Sensitivity = \frac{TP}{TP+FN} \end{aligned}$$

Accuracy measures the total number of correct classifications divided by the total number of cases. The formula is given as follows,

5 $$\begin{aligned} Accuracy = \frac{TP+TN}{TP+TN+FP+FN} \end{aligned}$$

### Training and test sets

Our dataset contained 5046 studies from 5046 patients. Data were randomly split into training and testing to avoid overlap between subjects. The 80% of the data was allocated to the training set, while 20% was allocated to the testing set. The training set includes 4036 subjects, while the remaining data of 1010 subjects was used for testing.

### Exploratory data analysis

In this work, we applied Exploratory Data Analysis (EDA) on dataset of radiological reports from the Breast Radiology department, TecSalud hospitals (Monterrey, Mexico) database. We used different graphical and statistical tools, like bar graphs, pie charts and word clouding methods, to examine the distribution of data set in different ways. The results of EDA provided insights into the structure and quality of the data set and the potential factors influencing radiological outcomes. The EDA also helped us selecting appropriate statistical models and hypotheses for further analysis.

**Fig. 3:**
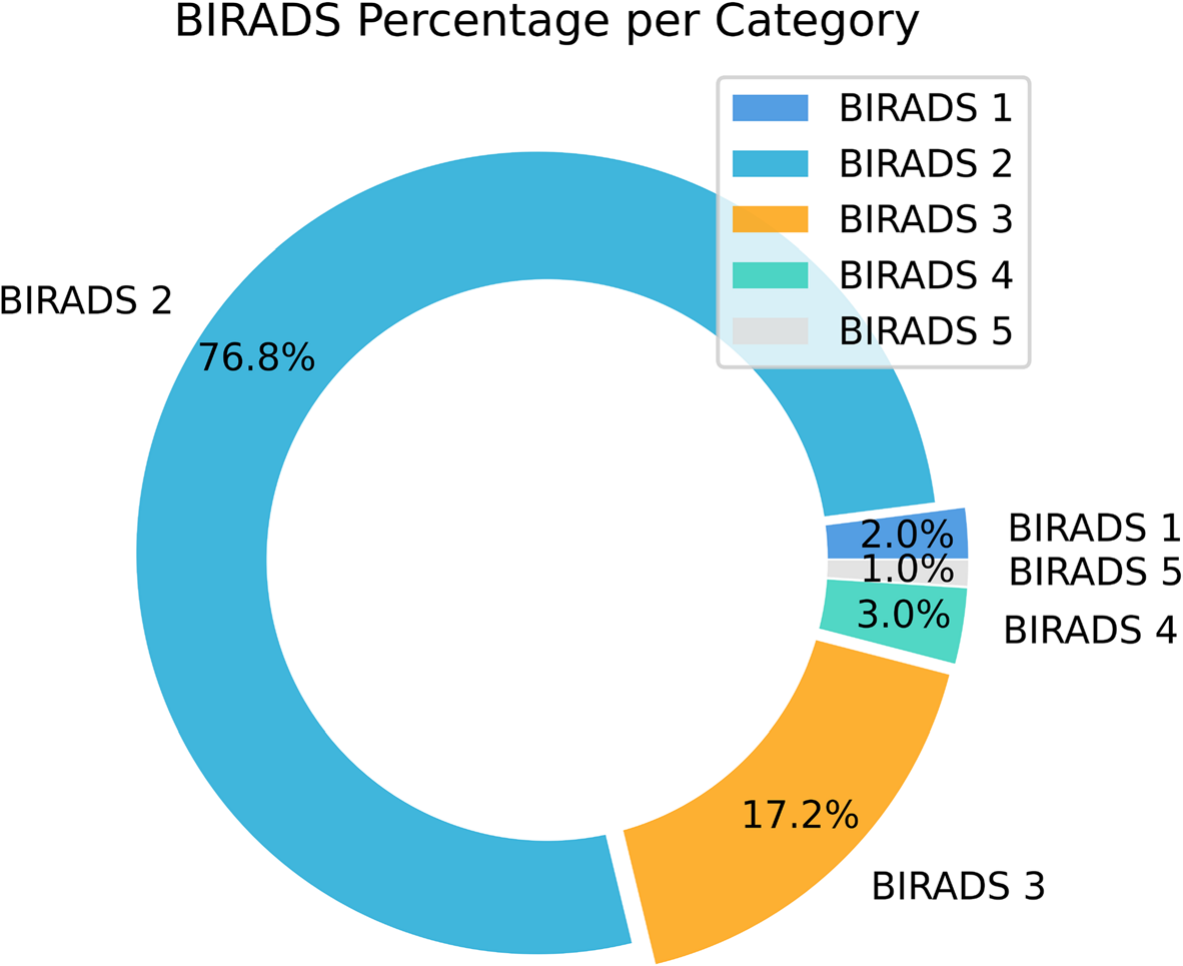
Overview of the BIRADS percentage in the dataset

**Fig. 4:**
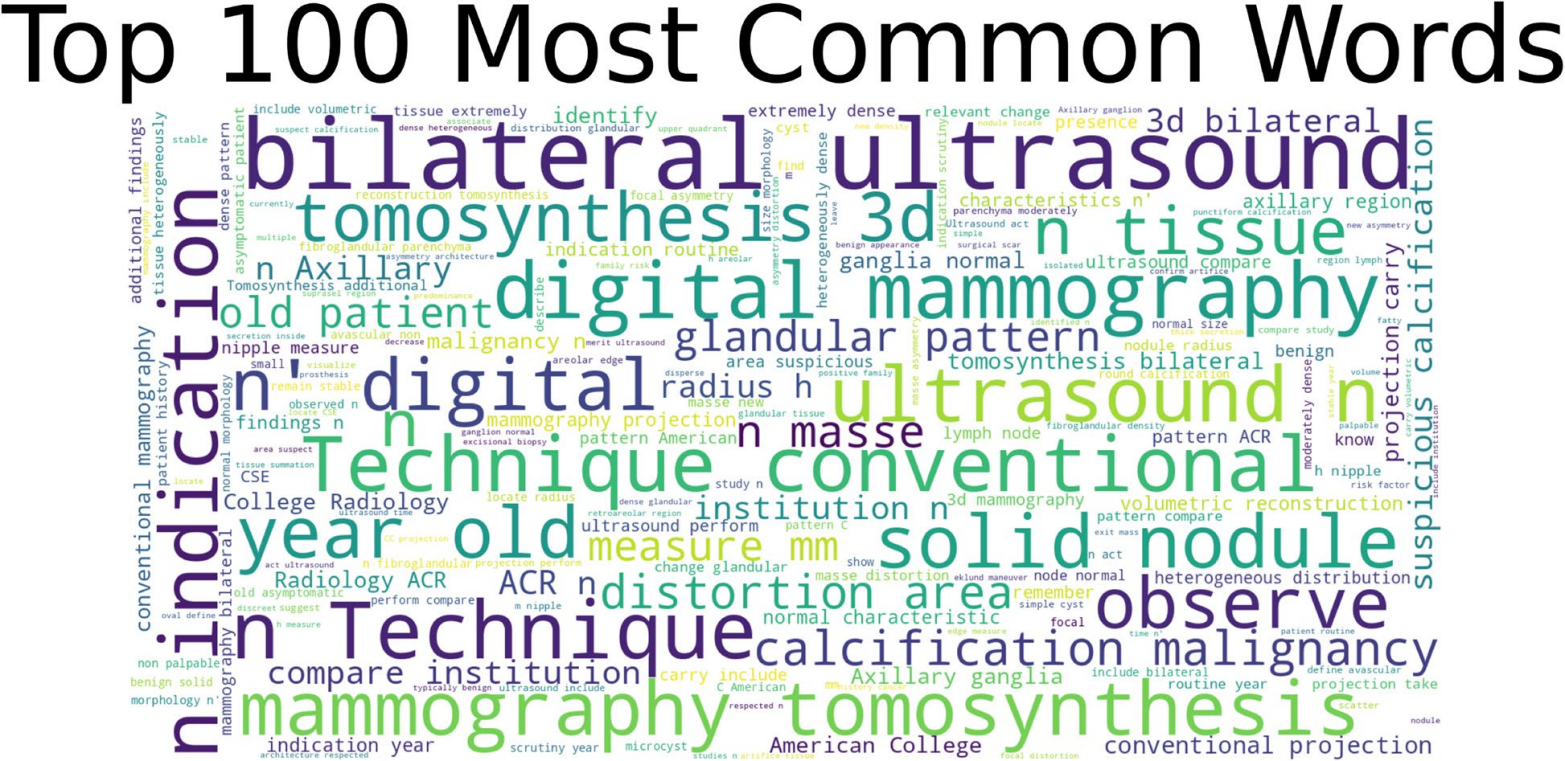
Representation of 100 most common words in our dataset using word clouding

To understand the distribution of each BIRADS category better, we have shown the BI-RADS distribution and percentage in Fig. 3. Moreover, an average number of letters and words per BI-RADS distribution is shown in Figure. S1 and Figure. S2 (Supplementary material), respectively. Furthermore, we used the word clouding technique to represent the 100 most common words in Fig. 4 in the dataset to understand the data further. Ultimately, in order to get further insights from each BI-RADS category (1,2,3,4,5), we have shown the frequency of the 25 most common words in Figures S3, S4, S5, S6 and S7 (Supplementary material), respectively.

## Results

Evaluation results are presented in Table 2, where we report the mean sensitivity and accuracy for each model with 95% CI using TF-IDF and word2vec as word embedding techniques on unpreprocessed and preprocessed data.

We used TF-IDF and word2vec as a word vectorizer to convert text into associated vectors to extract desired keywords and embeddings. We applied TF-IDF and word2vec on the data that was preprocessed and the one that was not preprocessed. We used mean sensitivity and accuracy as evaluation metrics to measure the performance across all the models.

The KNN classifier using TF-IDF achieved mean sensitivity and accuracy scores of 0.34 and 0.81, respectively, on unprocessed data, and 0.36 and 0.80 on preprocessed data. In contrast, using Word2Vec, it achieved 0.33 and 0.78 on unprocessed data, and 0.30 and 0.78 on preprocessed data. The SVM classifier using TF-IDF achieved mean sensitivity and accuracy scores of 0.44 and 0.85, respectively, on unprocessed data, and 0.42 and 0.85 on preprocessed data. When using Word2Vec, it obtained 0.20 and 0.77 on both unprocessed and preprocessed data. For the NB classifier, using TF-IDF resulted in sensitivity and accuracy scores of 0.20 and 0.78, respectively, on unprocessed data, and 0.22 and 0.79 on preprocessed data. Using Word2Vec, the scores were 0.20 and 0.76 on unprocessed data, and 0.24 and 0.70 on preprocessed data.

The RF classifier using TF-IDF achieved mean sensitivity and accuracy scores of 0.34 and 0.81, respectively, on both unprocessed and preprocessed data. With Word2Vec, the scores were 0.20 and 0.77, consistent across both unprocessed and preprocessed data. Using TF-IDF, the AdaBoost classifier achieved sensitivity and accuracy scores of 0.33 and 0.41, respectively, on unprocessed data, and 0.33 and 0.74 on preprocessed data. When using Word2Vec, the scores were 0.33 and 0.65 on unprocessed data, and 0.35 and 0.61 on preprocessed data. The GB classifier using TF-IDF achieved mean sensitivity and accuracy scores of 0.43 and 0.84, respectively, on unprocessed data, and 0.45 and 0.85 on preprocessed data. Using Word2Vec, it achieved 0.30 and 0.79 on unprocessed data, and 0.31 and 0.79 on preprocessed data. The XGB classifier using TF-IDF achieved mean sensitivity and accuracy scores of 0.49 and 0.85, respectively, on unprocessed data, and 0.52 and 0.86 on preprocessed data. Using Word2Vec, it achieved 0.33 and 0.80 on both unprocessed and preprocessed data.

The LSTM classifier achieved mean sensitivity and accuracy scores of 0.42 and 0.70, respectively, on unprocessed data, and 0.53 and 0.78 on preprocessed data. The BERT classifier achieved scores of 0.40 and 0.72 on unprocessed data, and 0.54 and 0.79 on preprocessed data. Finally, the BioGPT classifier achieved mean sensitivity and accuracy scores of 0.45 and 0.74 on unprocessed data, and 0.60 and 0.80 on preprocessed data.

From Table 2, it can be observed that BioGPT performs the best among all models on BI-RADS correct category classification in terms of mean sensitivity and XGB performed best in terms of accuracy. On the other hand, SVM and RF performed worse than any other model.

**Table 1:** The dataset comprises data from 5046 female participants of Mexican descent. It includes demographic details such as age and medical history, including breast implant status, prior cancer history, and previous surgeries or biopsies. Furthermore, it contains information on family cancer history, breast density classifications (A, B, C, D), and BI-RADS assessments across five categories. The dataset also records biopsy recommendations and confirmed cancer cases, as well as the mean time to a cancer event

Characteristics of dataset	
No. of Examples	5046
Sex	Women(5046), 100%
Race	Mexican Population
Age (Mean, SD, Range)	53, 9.99, 25-90
Patients Implants	No: 4185, Yes: 861
History of Previous Cancer	No: 4525, Yes: 521
Previous Surgeries/Biopsies	No: 4160, Yes: 886
Family History of Cancer	No: 4296, Yes: 750
Breast Density Distribution	A: 641, B: 1271, C: 2053, D: 1064
Distribution of BIRADS	1: 117, 2: 3921, 3: 802, 4: 129, 5: 77
Biopsy Recommendation	No: 4796, Yes: 250
Patients with Confirmed Cancer	30
Cancer Development in Five Years	61
Mean Time to Cancer Event	Mean: 1000.008 Days, SD: 898.91

**Table 2:** This table presents detailed results from seven machine learning models and three deep learning models. The first section outlines the performance of the machine learning models, reporting mean sensitivity and accuracy using two word embedding techniques: Word2Vec and TF-IDF. Confidence intervals are provided alongside the sensitivity and accuracy scores, with results shown separately for both unprocessed and preprocessed data. The second section details the performance of the deep learning models, also reporting sensitivity, accuracy, and their corresponding confidence intervals for both preprocessed and unprocessed data

**Machine learning methods**								
**Word embs.**	**Word2Vec**				**TF-IDF**			
**Model**	**U-Data (mSen.)**	**P-Data (mSen.)**	**U-Data (mAcc.)**	**P-Data (mAcc.)**	**U-Data (mSen.)**	**P-Data (mSen.)**	**U-Data (mAcc.)**	**P-Data (mAcc.)**
KNN	0.33	0.30	0.78	0.78	0.34	0.36	0.81	0.80
	(0.238-0.422)	(0.210-0.389)	(0.755-0.806)	(0.757-0.808)	(0.247-0.433)	(0.266-0.454)	(0.792-0.840)	(0.782-0.830)
SVM	0.20	0.20	0.77	0.77	0.44	0.42	0.85	0.85
	(0.122-0.278)	(0.122-0.278)	(0.750-0.801)	(0.750-0.801)	(0.343-0.537)	(0.323-0.517)	(0.837-0.880)	(0.835-0.878)
NB	0.20	0.24	0.76	0.70	0.20	0.22	0.78	0.79
	(0.122-0.278)	(0.156-0.324)	(0.738-0.791)	(0.673-0.729)	(0.247-0.433)	(0.266-0.454)	(0.787-0.835)	(0.789-0.837)
RF	0.20	0.20	0.77	0.77	0.34	0.36	0.81	0.81
	(0.122-0.278)	(0.122-0.278)	(0.750-0.801)	(0.750-0.801)	(0.247-0.433)	(0.266-0.454)	(0.787-0.835)	(0.789-0.837)
AdaBoost	0.33	0.35	0.65	0.61	0.33	0.33	0.41	0.74
	(0.238-0.422)	(0.256-0.443)	(0.627-0.686)	(0.589-0.649)	(0.238-0.422)	( 0.238-0.422)	(0.389-0.450)	(0.715-0.769)
GB	0.30	0.31	0.79	0.79	0.43	0.45	0.84	0.85
	(0.210-0.389)	(0.219-0.400)	(0.773-0.823)	(0.774-0.824)	(0.333-0.527)	(0.352-0.547)	(0.818-0.863)	(0.829-0.872)
XGB	0.33	0.33	0.80	0.80	0.49	0.52	0.85	0.86
	(0.238-0.422)	(0.238-0.422)	(0.778-0.828)	(0.784-0.832)	(0.392-0.588)	(0.422-0.618)	(0.838-0.881)	(0.840-0.883)
**Deep learning methods**								
**Model**		**U-Data(mSen)**		**P-Data(mSen)**		**U-Data(Accuracy)**		**P-Data(Accuracy)**
LSTM		0.42		0.53		0.70		0.78
		(0.346-0.490)		(0.455-0.619)		(0.673-0.730)		(0.753-0.805)
BERT		0.40		0.54		0.72		0.79
		(0.331-0.459)		(0.477-0.607)		(0.692-0.746)		(0.768-0.819)
BioGPT		0.45		**0.60**		0.74		0.80
		(0.235-0.669)		(0.391-0.811)		(0.710-0.764)		(0.772-0.822)

## Discussion

Numerous studies have been conducted on information extraction from radiological reports [30, 33–41] but only a few studies have released datasets on radiological reports and baselines [42–46]. Furthermore, a few studies have focused on structured information extraction from breast imaging-based radiological reports [12, 47–49], however, the data has not been released publicly. It has been shown in the literature that AI based breast cancer diagnosis can be greatly enhanced by the information extracted from the patient radiological reports, however, lack of public dataset has restricted further research into this domain. In order to address this gap, we have curated and make the radiological report based dataset available to drive further research in this direction.

In this work, we compared ML, DL and LLMs-based architectures. We evaluated the model’s performance using mean sensitivity and accuracy. We observed that the LLM i.e., BioGPT model achieved best mean sensitivity of 0.60, which is 6% higher than the second best classifier BERT. In terms of accuracy, XGB performed best compared to all other models with an accuracy of 0.86.

In this work, we explored two word embedding techniques with ML-based classifiers to extract relevant information. We also limit our work to only extracting BI-RADS category classification. In future, we plan to extend the dataset and add associated mammography images while applying vision-language models to analyze the performance. Also, we plan to classify various other factors necessary for breast cancer diagnosis and prognosis, such as benign and malignant cases, age of the patient, family history of the cancer, risk of the cancer, recurrence, breast density and more.

Our benchmark will help and encourage the scientific community to work on extracting relevant information by applying SOTA ML, DL and LLMs architectures that will lead to rapid improvement in radiological language processing.

## Conclusion

In conclusion, we curated and annotated a new breast imaging-based radiological reports dataset. This dataset consists of 5,046 radiological reports. These reports are based on mammography, DBT and breast US. We also compared the baseline performance of ML, DL and LLMs architectures on the dataset. This study used TF-IDF and word2vec as word embedding techniques. The BioGPT classifier with preprocessed text performed better with a mean sensitivity of 0.60, compared to all the other classifiers using TF-IDF and word2vec word embedding techniques. In terms of accuracy, XGB outperformed all the other classifiers by achieving a score of 0.86. Our work provides baselines on TECRR dataset for the researchers and clinicians for further investigation. It can improve the classification accuracy using different ML, DL and LLMs based techniques.

## Supplementary Information

Supplementary Material 1

## Data Availability

All papers are available on publisher websites. All data generated or analyzed during this study are included in this published article. The dataset underlying this article will be shared on reasonable request to the corresponding author.

## Abbreviations

TF-IDF: Term Frequency Inverse Document Frequency
GB: Gradient Boosting
MRI: Magnetic resonance imaging
US: Ultrasound
DBT: Digital breast tomosynthesis
DL: Deep learning
ML: Machine learning
NLP: Natural language processing
DM: Digital mammography
BI-RADS: Breast Imaging Reporting and Data System
ACR: American College of Radiology
