# Supplementary material for: TECRR: a benchmark dataset of radiological reports for BI-RADS classification with machine learning, deep learning, and large language model baselines
Source: BMC Med Inform Decis Mak. 2024 Oct 24;24:310. doi: 10.1186/s12911-024-02717-7 (PMC11515610; doi:10.1186/s12911-024-02717-7)

Supplementary Figurers:

Figure S1: Average Number of Letters per BIRADS Distribution

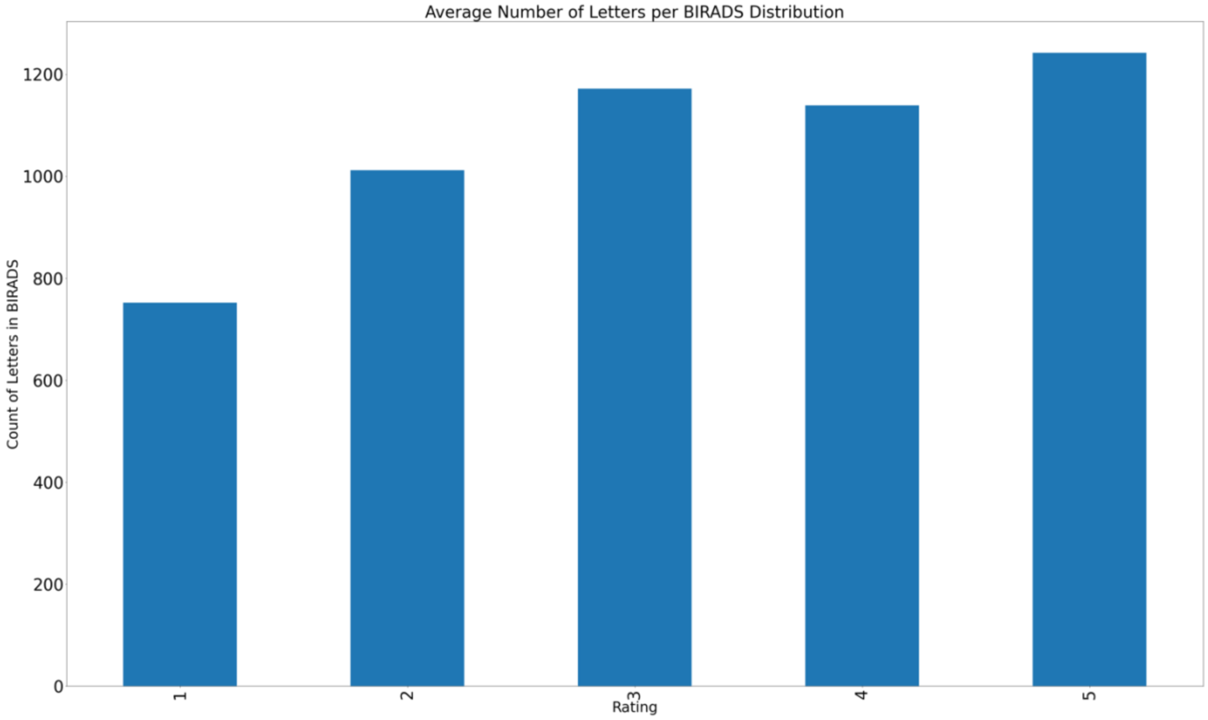

Figure S2: Average Number of Words per BIRADS Distribution

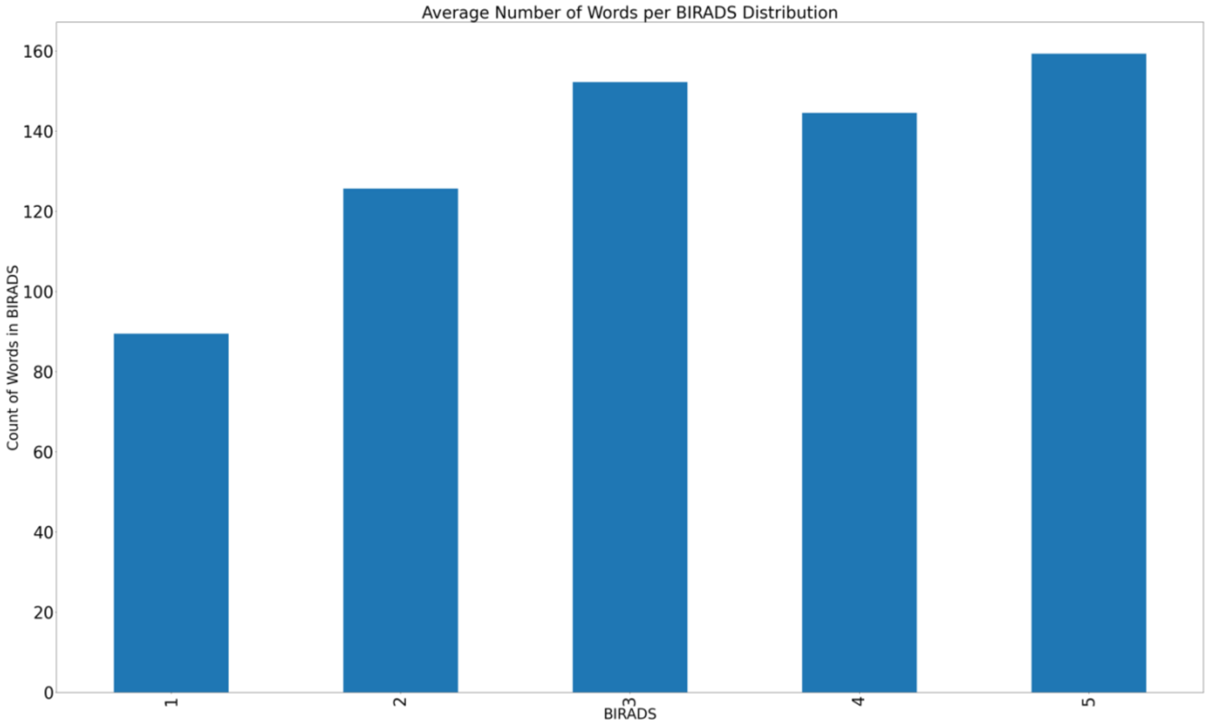

Figure S3: Frequency of 25 Most Common Words for BIRADS 1

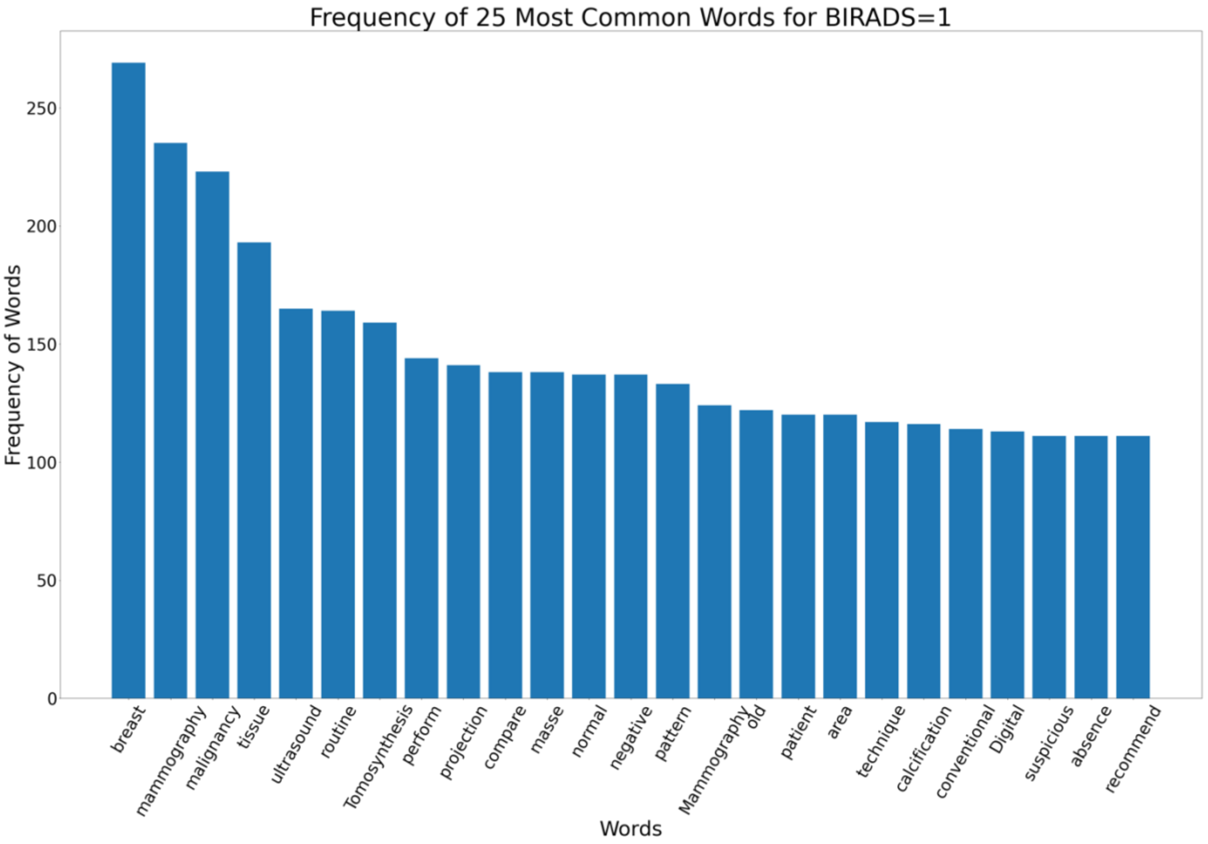

Figure S4: Frequency of 25 Most Common Words for BIRADS 2

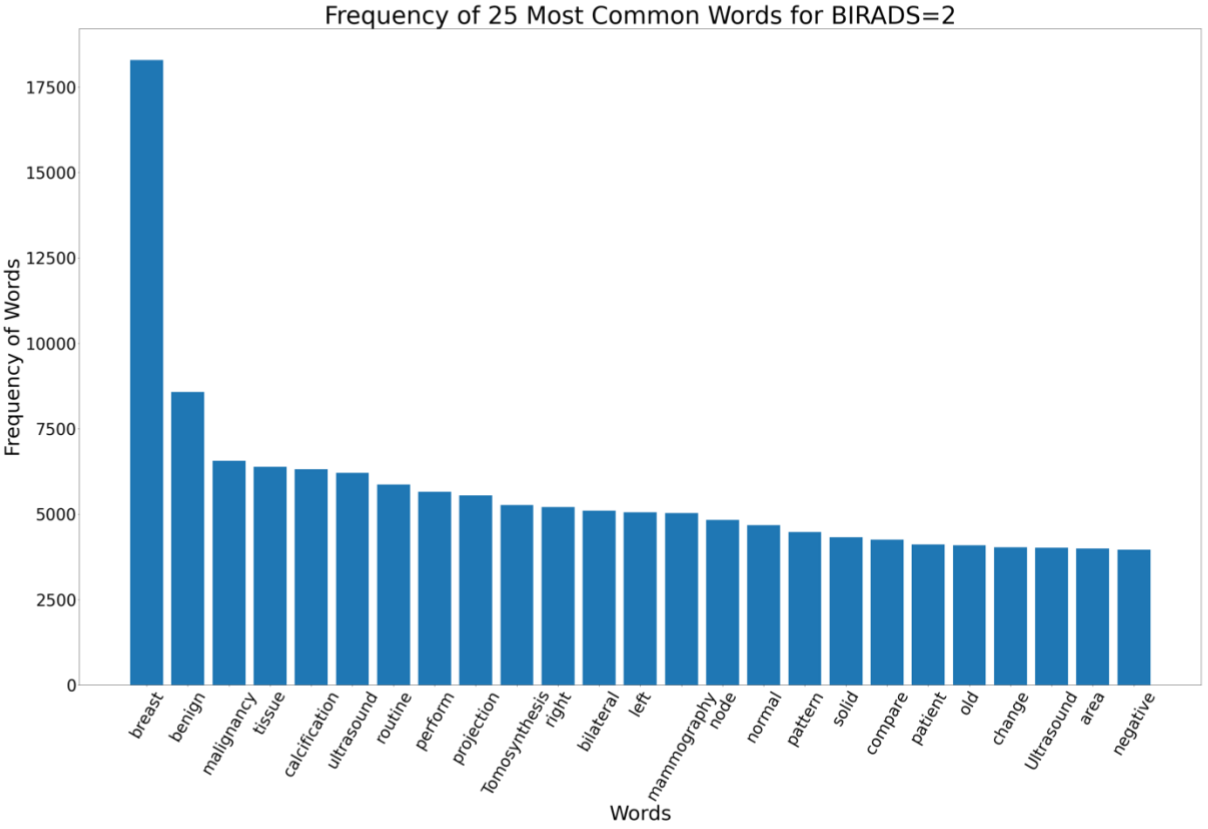

Figure S5: Frequency of 25 Most Common Words for BIRADS 3

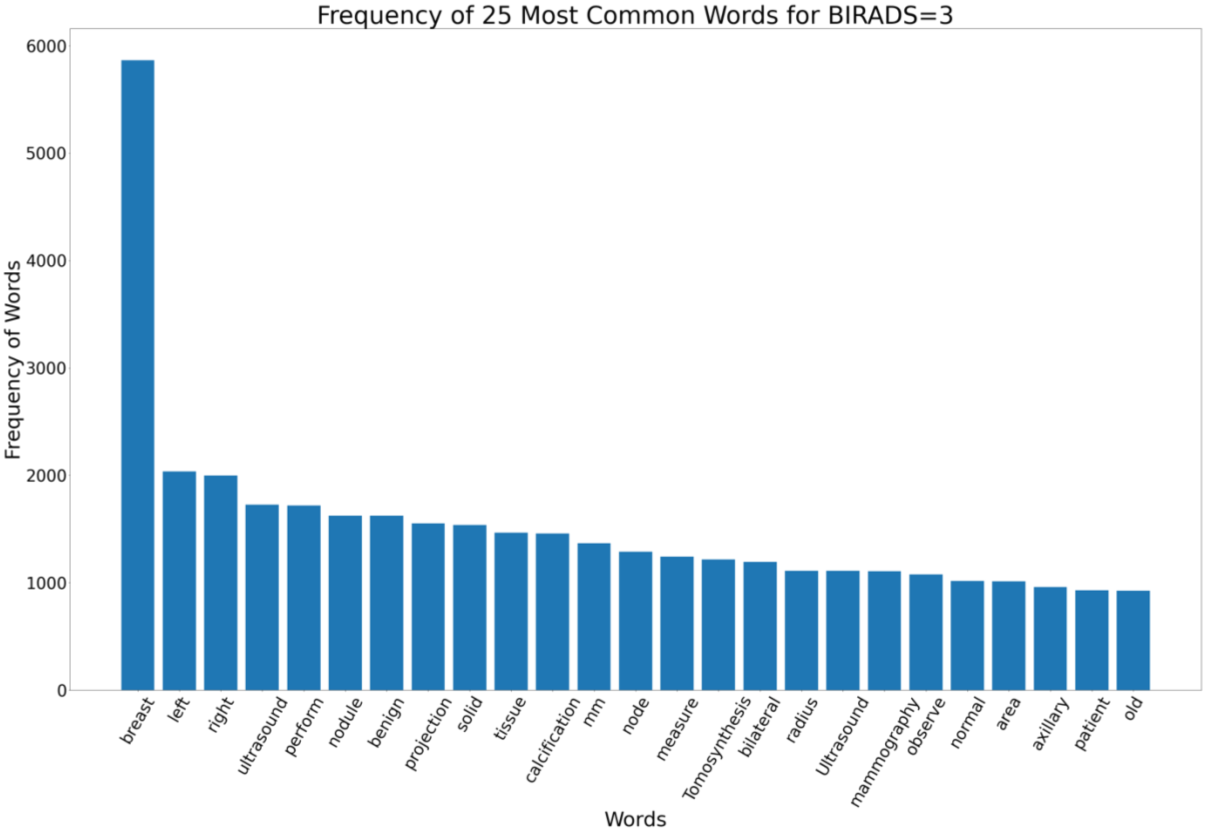

Figure S6: Frequency of 25 Most Common Words for BIRADS 4

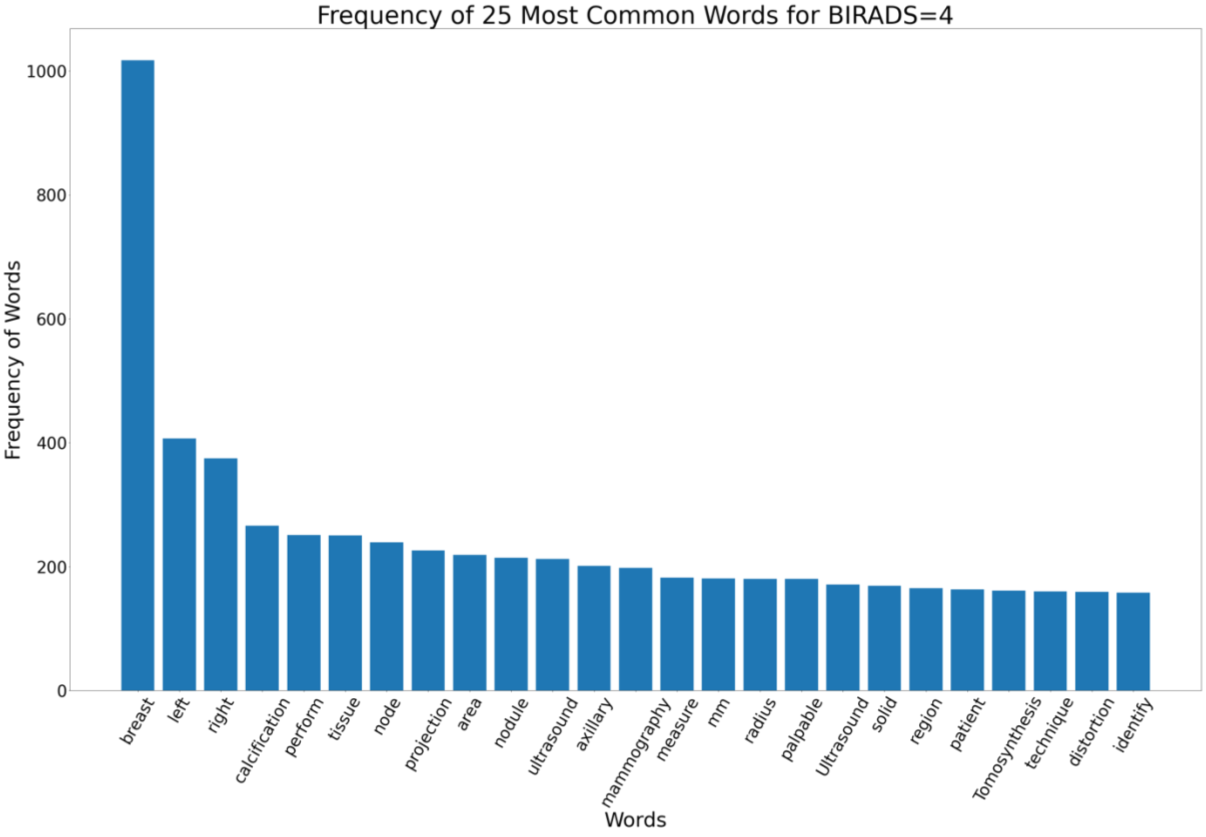

Figure S7: Frequency of 25 Most Common Words for BIRADS 5

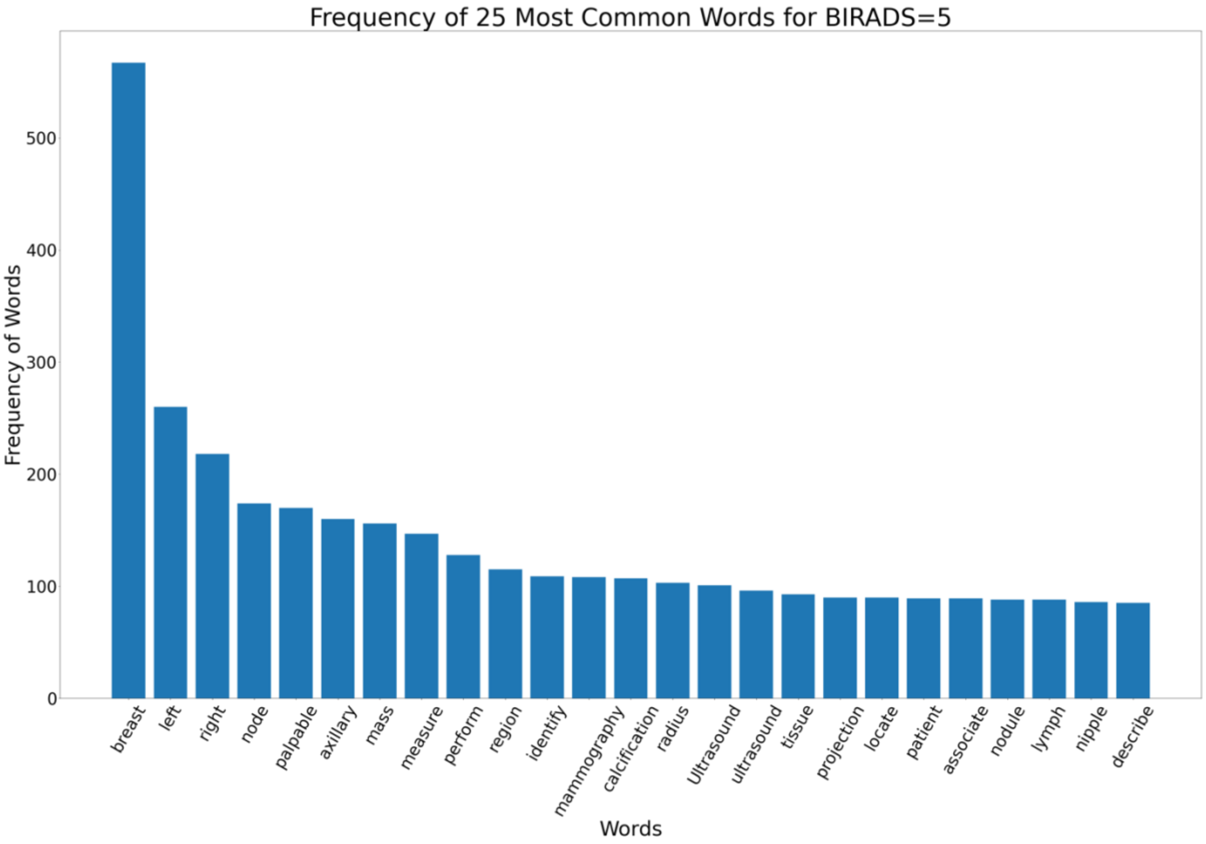

Supplement: Supplementary file 1 — Supplementary Material 1 [file 12911_2024_2717_MOESM1_ESM.pdf]
